# Coevolution between a Family of Parasite Virulence Effectors and a Class of LINE-1 Retrotransposons

**DOI:** 10.1371/journal.pone.0007463

**Published:** 2009-10-15

**Authors:** Soledad Sacristán, Marielle Vigouroux, Carsten Pedersen, Pari Skamnioti, Hans Thordal-Christensen, Cristina Micali, James K. M. Brown, Christopher J. Ridout

**Affiliations:** 1 Department of Disease and Stress Biology, John Innes Centre, Norwich, United Kingdom; 2 Computational and Systems Biology Department, John Innes Centre, Norwich, United Kingdom; 3 Department of Agriculture and Ecology, Faculty of Life Sciences, University of Copenhagen, Copenhagen, Denmark; 4 Department of Plant-Microbe Interactions, Max-Planck Institute for Plant Breeding Research, Köln, Germany; University of Hyderabad, India

## Abstract

Parasites are able to evolve rapidly and overcome host defense mechanisms, but the molecular basis of this adaptation is poorly understood. Powdery mildew fungi (Erysiphales, Ascomycota) are obligate biotrophic parasites infecting nearly 10,000 plant genera. They obtain their nutrients from host plants through specialized feeding structures known as haustoria. We previously identified the *AVR*_k1_ powdery mildew-specific gene family encoding effectors that contribute to the successful establishment of haustoria. Here, we report the extensive proliferation of the *AVR*_k1_ gene family throughout the genome of *B. graminis*, with sequences diverging in *formae speciales* adapted to infect different hosts. Also, importantly, we have discovered that the effectors have coevolved with a particular family of LINE-1 retrotransposons, named TE1a. The coevolution of these two entities indicates a mutual benefit to the association, which could ultimately contribute to parasite adaptation and success. We propose that the association would benefit 1) the powdery mildew fungus, by providing a mechanism for amplifying and diversifying effectors and 2) the associated retrotransposons, by providing a basis for their maintenance through selection in the fungal genome.

## Introduction

There is strong selection pressure on parasites to develop strategies to successfully infect whilst evading host detection and defense mechanisms [1]. Important components of the pathogenicity arsenal of parasites are effectors, usually secreted proteins that influence host metabolism or defense mechanisms to provide an environment for successful infection [2]. Resistance (*R*) genes are part of the plant defense system, and are widely used in agriculture to control parasites. Most of the known *R* genes encode nucleotide binding site leucine rich repeat (NBS-LRR) receptors [1]. When an NBS-LRR protein recognizes specific parasite avirulence (AVR) molecules, plant defense responses that prevent further infection are induced in accordance with the gene-for-gene (GFG) model [3]. Some bacterial and oomycete AVR proteins are known to be effectors, but little is known about the function of most fungal AVR molecules [2], [4]. Parasites may evolve to overcome host resistance by altering their *AVR* genes to avoid *R*-dependent recognition [1], [5], [6].

GFG resistance has been extensively investigated in the interaction between barley and barley powdery mildew (*Blumeria graminis* f. sp. *hordei, Bgh*), an obligate fungal parasite. More than 85 barley *R* genes, including 28 alleles at the *Mla* locus, have been described, each conferring resistance to *Bgh* isolates with matching *AVR* genes [7]. Mla proteins are nucleotide binding site leucine rich repeat (NBS-LRR) receptors. They share >90% amino acid sequence identity but recognise isolate-specific *Bgh AVR* gene products [8]. More than 25 independent *AVR* gene loci have been described in *Bgh* isolates [9], [10], and genetic crosses have shown that genes for up to eight linked *AVR* specificities are clustered at a complex set of loci [11], [12]. *B. graminis* exhibits a high level of host specialization and eight *formae speciales* (*ff. spp*.) infecting cereals and forage grasses are known [13], [14]. The genetic basis for such host specialization is as yet unknown, but several genes are likely to be involved [15].

We previously isolated *AVR*_k1_ (Q09QS2) and *AVR*_a10_ (Q09QS3) genes which, when present in *Bgh* isolates, induce resistance in barley lines containing *Mlk1* and *Mla10* genes, respectively [16]. We also provided the first evidence that these fungal *AVR* genes encode effectors that contribute to the establishment of haustoria, the essential feeding structures of *Bgh* [16]. The predicted amino acid sequences of *AVR*_k1_ and *AVR*_a10_ do not contain signal peptides, indicating that they are not secreted from the parasite in the same way as the majority of known fungal and oomycete AVR proteins [17], [18]. When expressed in barley cells, AVR_a10_ induces an association between Mla10 and a WRKY-2 transcription factor in the nucleus, which may initiate defense gene activation [19]. *AVR*_k1_ and *AVR*_a10_ belong to a family of closely-related paralogs (hereafter called *AVR*_k1_ family or *AVR*_k1_ paralogs) which encode proteins with a core domain of conserved amino acids [16].

Some parasite effector genes are found in the proximity of transposable elements (TEs), which have been postulated to provide a mechanism for their expansion and movement within and among genomes [5], [6]. Some transposon insertions into *AVR* gene loci have resulted in the loss of avirulence (i.e. gain of virulence on hosts with specific resistance genes) of bacterial and fungal parasites [16], [20]–[22]. We previously demonstrated that members of the *AVR*_k1_ family lie close to TE1a LINE-1 retrotransposons (RTs), and both sequences can be expressed as a single transcript [12], [16]. Here, we report the extensive proliferation of the *AVR*_k1_ gene family throughout the genome of *B. graminis*, with sequences diverging in *ff. spp.* adapted to infect different hosts. Furthermore we show that the *AVR_k_*_1_ family has coevolved with the lineage of TE1a RTs, suggesting a mutual advantage from the association which may ultimately benefit parasite adaptation and success.

## Results

### The *AVR*_k1_ effector gene family is unique to powdery mildew fungi

**Figure 1:**
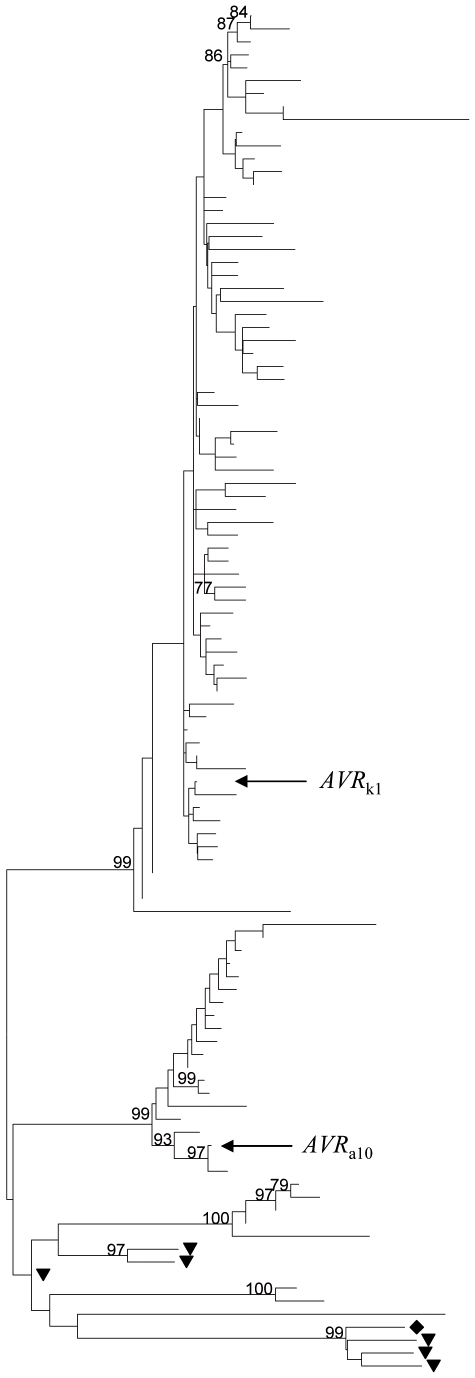
Neighbor-joining consensus tree showing the relationship between *AVR*_k1_ homologs from powdery mildew genomes. *B. graminis* sequences were retrieved from an nrdb90 database as described in the text; near-identical sequences were removed for clarity. The figure shows 105 amino acid sequences, including *AVR*_k1_, *AVR*_a10_ and 96 ORFs predicted from *Bgh*, six ORFs predicted from the *Erysiphe pisi* genome (marked with a triangle) and one ORF predicted from the *Erysiphe* (*Golovinomyces*) *orontii* genome (the closest homologue to *AVR_k1_* of the six found, marked with a diamond). Bootstrap support (1,000 replicates) is shown if higher than 70%.

An initial BLAST [23] of the draft *Bgh* genome sequence (http://www.blugen.org/), resulted in 1145 homologs to *AVR*_k1_ with Expect (E) values ranging from 7e^−62^ to 1e^−5^. To investigate the phylogenetic diversity of these paralogs, we created an nrdb90 database (non-redundant set of the predicted open reading frames with 90% sequence identity threshold). Proteins shorter than 100 residues were discarded. This search resulted in 260 sequences which were clearly paralogous to AVR_k1_ (including 94 paralogs of AVR_a10_) with Expect (E) values ranging from 1e^−152^ to 1e^−10^. Homologous sequences were also found in the genomes of the powdery mildew fungi *Erysiphe* (*Golovinomyces*) *orontii* (six homologs, 1e^−3^<E<1e^−8^), which infects *Arabidopsis thaliana*, and *Erysiphe pisi* (six homologs, 1e^−4^<E<1e^−17^), which infects pea. None of the *Erysiphe* sequences grouped in the clades containing *AVR*_k1_ or *AVR*_a10_ (Fig. 1). *AVR_k1_* or *AVR*_a10_ homologs were not found in BLAST searches (E value <1e^−5^) against the EMBL/GenBank [24], COGEME phytopathogen EST database [25], Broad Institute (Fungal Genome Initiative fungi) and Uniprot [26] databases, indicating that this gene family is specific to powdery mildew fungi.

### The *AVR*_k1_ gene family has diverged in accordance with *B. graminis* ff. spp. specialized on different hosts

**Figure 2:**
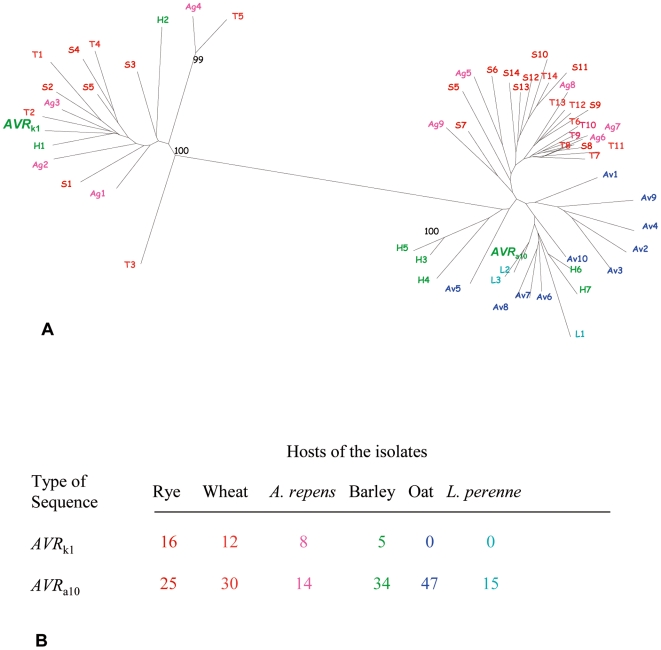
Analysis of sequences of the *AVR*_k1_ family from *formae speciales* of *B. graminis*. A. Neighbor Joining tree of the sequences obtained by degenerate primers from isolates of *B. graminis* from grass hosts: rye (f. sp. *secalis*, S, in red), wheat (f. sp. *tritici*, T, in orange), *Agropyron* spp. (f. sp. *agropyri*, Ag, in magenta), barley (f. sp. *hordei*, H, in green), oat (f. sp. *avenae*, Av, in blue) and *Lolium perenne* (L, in cyan). The sequences of the genes *AVR*_k1_ and *AVR*_a10_ are in a larger font. Bootstrap support (1,000 replicates) is shown if higher than 90%. Only sequences with a maximum identity to other sequences in the family less than 90% were used in the analysis. B. Number and type of sequences homologous to *AVR*_k1_ and *AVR*_a10_ obtained by degenerate PCR from *B. graminis* from different hosts.

On the basis of the known role of AVR_k1_ and AVR_a10_ proteins in pathogenicity, we predicted that sequences of *AVR*_k1_ paralogs might have diverged from each other in *B. graminis* isolates adapted to infect different host genera. To test this hypothesis, degenerate PCR primers designed from the conserved core of the AVR_k1_ and AVR_a10_ protein sequences were used to amplify genomic DNA and clone the corresponding gene regions from ff. spp. infecting cereal crops and the grasses *Elytrigia repens* (synonym *Agropyron repens*) and *Lolium perenne*. The sequences obtained were classified into two subfamilies: the *AVR*_k1_-like clade and the *AVR*_a10_-like clade (Fig. 2A). Nucleotide identity within subfamilies was very high, around 80%. The number of sequences in the sub-family which grouped with *AVR*_a10_ was four times higher than the number of *AVR*_k1_-like sequences. Moreover, the relative number of sequences of each type differed significantly depending on the host of each f. sp. (χ^2^ = 34.1, P<10^−3^; Fig. 2B). None of the sequences amplified from powdery mildew isolates of oats (f. sp. *avenae*) or *L. perenne* grouped with the *AVR*_k1_-like clade (Fig. 2A), indicating the absence or low abundance of this subfamily in these *ff. spp.*

The internal branches of both *AVR*_k1_-like and *AVR*_a10_-like clades were not supported statistically, possibly due to a phase of rapid divergence during expansion of the gene family [27]. Therefore, we used a likelihood mapping test [28] to examine if there was a relationship between the groups of sequences within each clade and the *f.sp.* from which they originated. There was no statistical support for any such grouping within the *AVR*_k1_-like clade. By contrast, an association between the *AVR*_a10_ sequences and *ff.spp.* was found: 91% of the quartets grouped the sequences from ff.spp. *tritici*, *secalis* and *agropyri* separately from the sequences from ff.spp. *avenae*, *hordei* and the isolate from *L. perenne* (Fig. 2A, Fig. S1). Therefore the *AVR*_a10_ sequences have diverged with the powdery mildew *formae speciales* infecting different Poaceae host genera.

### *AVR*_k1_ paralogs contain conserved and diversified regions

The very large number of *AVR*_k1_ paralogs detected in the *B. graminis* genome may not reflect the actual number of expressed genes. Indeed, many gene duplications can be subject to gene inactivation through mutation or deletion/insertion events as well as DNA methylation. To study the expressed *AVR*_k1_ paralogs, we analyzed the *B. graminis* transcriptome amplified by 5′ and 3′RACE RT-PCR. In total, 49 5′ RACE sequences and 84 3′RACE sequences were obtained from four isolates of f. sp. *hordei* and one isolate of f. sp. *tritici*, revealing considerable divergence in their length and degree of homology with *AVR*_k1_ (Table 1). The 3′RACE sequences were significantly less conserved than those obtained by 5′RACE (t-test for comparison of average nucleotide identities with *AVR*_k1_, P<10^−14^).

**Table 1:** Expressed paralogs of *AVR*_k1_ from the different isolates of *B. graminis*.

		RACE 5′			RACE 3′		
*Forma specialis*	Isolate	No of different seq.	Length	% Identity to *AVR*_k1_	No of different seq.	Length	% Identity to *AVR*_k1_
*hordei*	A6	6	417±291	83±16	13	724±501	45±13
	CC52	7	560±34	87±1	14	205±70	73±13
	CC148	11	422±178	82±5	18	695±527	58±16
	DH14	12	417±204	78±17	19	578±354	53±14
*tritici*	JIW11	13	466±165	86±7	20	150±65	75±9

The table shows number and mean ± standard deviation of the lengths (bp) and percentage of nucleotide identity to *AVR*_k1_ of the sequences obtained by 5′ and 3′RACE PCR.

Several parasite effectors are under diversifying selection (DS), evolving rapidly to avoid immune detection systems within the host [2]. We tested for DS in a set of 113 *AVR*_k1_ paralogs obtained by RACE RT-PCR. We used a maximum likelihood method to identify specific amino acid residues that are under positive selection (with a nonsynonymous/synonymous rate ratio higher than one, ω = d_N_/d_S_ >1) [29]. Most analyzed residues in the core region of the expressed *AVR*_k1_ paralogs are under purifying selection. This indicates a high level of sequence conservation, possibly due to protein functional or structural constraints. DS was evident in a region immediately 5′ to the core. This indicates that this region is evolving rapidly, so it could be involved in adaptation to avoid *R* gene recognition, as proposed for *Phytophthora* effectors [30] (Fig. S2A). By comparing complete cDNAs, breakpoints of nucleotide divergence could be identified shortly after the sequence homologous to the *AVR*_k1_ protein (Fig. S2B and S3A, B). This suggests that *AVR*_k1_ sequence proliferation has occurred through gene duplication and insertion at several distinct sites within the *Bgh* genome.

### *AVR*_k1_ paralogs are associated with TE1a retrotransposons

Of the 17 3′RACE sequences longer than 800 nucleotides, 65% had homology with retrotransposons (RTs) at their 3′end, increasing to 90% for sequences longer than 1200 nucleotides. Most (10/11) of the predicted homologies had an amino acid identity of 70–80% with the nucleic acid binding domain of *Bgh* TE1a RTs that we reported previously [12], [16]. Full-length sequences were also obtained by hybridization to a cDNA library, with similar results. Four of 22 full-length cDNA clones were natural antisense transcripts [NATs, 31] with a polyT tail at the 5′ end before the ATG translation start site. The genomic region containing the NATs was identified by BLAST with the draft *Bgh* genomic sequence. The presence of polyT at the 5′ end of the cDNA sequences confirms that the sequences are transcribed in the reverse orientation (Fig. S4).

We further investigated the association between the *AVR*_k1_ gene family and RTs, by testing the extent to which TE1a and *AVR*_k1_ predicted open reading frames occurred together in the draft *Bgh* genome sequence. Three categories of hits were identified: 1) ‘Common’ hits were those in which *AVR*_k1_ and TE1a sequences occurred in the same open reading frame. 2) ‘Adjacent’ hits were those in which *AVR*_k1_ and TE1a sequences occurred on the same contig but were separated by a stop codon. Pairs were not considered adjacent if one hit was on the complementary strand. Additionally, we specified that each member of a pair could only belong to a maximum of one pair. 3) ‘Unique’ hits matched a specific contig containing either *AVR*_k1_ or TE1a paralogs, but not both. We found that 57.8% of *AVR*_k1_ paralogs were either ‘common’ or ‘adjacent’ to TE1a homologs. This proportion is significantly higher than the proportion of TE1a homologs found common or adjacent to the two largest *Bgh* gene families other than *AVR*_k1_ (Table 2, χ^2^ test, P<10^−4^). Conversely, the proportion of TE1a homologs common or adjacent to *AVR*_k1_ paralogs was significantly higher than the proportion found with the four largest families of repetitive elements other than TE1a (Table 3, χ^2^ test, P<10^−4^). These two results demonstrate that there is a significant association between *AVR*_k1_ and TE1a sequences.

**Table 2:** TE1a sequences are associated with *AVR*_k1_ paralogs, and no other gene families.

Gene family	Repetitive element	Number of common hits	Number of adjacent hits	Number of hits with no repeat neighbor	Total number of homologs
*AVR*_k1_	TE1a	532	130	483	1145
	EGH24	1	154	990	
A8U3S4	TE1a	0	18	405	423
	EGH24	0	57	366	
Q9HGU6	TE1a	0	1	57	58
	EGH24	0	0	58	

Common, adjacent and unique hits of paralogs of three *Bgh* gene families with TE1a and EGH24 repetitive elements. A8U3S4: kinase transferase, Q9HGU6: kinase transferase. Cut off for homologies: E≤10^−5^.

**Table 3:** *AVR*_k1_ paralogs are associated with TE1a, and not other classes of repetitive sequence.

Repetitive element	Number of common hits	Number of adjacent hits	Number of hits with no *AVR*_k1_ neighbor	Total number of homologs
TE1a	532	130	1085	1747
EGH24	1	154	4274	4429
Q5BBQ3	0	50	2008	2058
Q9ZT24	0	43	1927	1970
Q2AA50	0	46	1747	1793

Common, adjacent and unique hits of paralogs of five *Bgh* repetitive elements with *AVR*_k1_. EGH24: SINE-like repetitive element, Q9ZT24: aspartyl protease hydrolase, Q2AA50: retrotransposon gag protein. Q5BBQ3; reverse transcriptase. Cut off for homologies: E≤10^−5^.

We examined which other sequences were found in the proximity of the 483 *AVR*_k1_ homologs that were not situated next to TE1a sequences (Table 2). We retrieved 10 kb-long contig sequences (5 kb either side of the hit), fragmented them into 2 kb segments (each overlapping by 1 kb) and searched for sequence homology of each fragment establishing a cut-off of E≤10^−5^. A total of 59 different proteins were found. Fifty three of them had homologs that appeared 10 times or less (31 appeared only once, which means that no homolog was found for these particular genes). The sequences most commonly found close to these *AVR*_k1_ sequences were TE1a sequences (284 hits), followed by another retrotransposon family, TE1b (192 hits, Table 4). Therefore, no other type of sequence is associated with the *AVR*_k1_ family.

**Table 4:** TE1a is the gene family most frequently situated in the proximity of *AVR*_k1_ homologs.

Gene family^a^	Number of homologs	Putative function/homology with other sequences
A8U3R5	12	Hypothetical protein
Q8S7A3	32	Putative retroelement
A8U3S6	38	TE3 retrotransposon
A8U3R2	192	TE1b retrotransposon
A8U3R0	284	TE1a retrotransposon

Number of homologs and putative function of the gene families containing more than 10 members situated in the proximity of any of the 483 *AVR*_k1_ paralogs that were not associated to TE1a sequences.

a Gene families defined by all homologs found with a cut-off E≤10^−5^. The names of the gene families correspond to the top hits.

We investigated if associations between retrotransposable elements and gene families are common events in the *Bgh* genome. We searched for cases where the most frequent repetitive element found in *Bgh* genome (EGH24) occurred close to other gene families. We did not find any case with a proportion of common or adjacent hits equivalent to that found with TE1a and *AVR*_k1_ paralogs (Table 2). To further test if other types of sequence could be associated with TE1a homologs, we examined the 1085 TE1a hits that were neither common nor adjacent to *AVR*_k1_ paralogs (Table 3) with the same procedure used for *AVR*_k1_ explained above. A total of 112 different proteins were found, of which 101 had homologs that appeared 10 times or less. The family that was most commonly found close to TE1a sequences was a reverse transcriptase (1415 hits). The other most frequent families were Gag-like or reverse transcriptases, typical of retrotransposons (Table 5). Therefore apart from the *AVR*_k1_ family, only retrotransposable elements are frequently found in the proximity of TE1a sequences.

**Table 5:** Only retrotransposon sequences, other than *AVR*_k1_ paralogs, are frequently situated in the proximity of TE1a homologs.

Gene family^a^	Number of homologs	Putative function/homology with other sequences
Q2GV21	20	Hypothetical protein
A8U3R5	39	Hypothetical protein
Q9C436	51	Gag protein
A4QX15	74	Hypothetical protein
Q2HI73	105	Hypothetical protein
Q7XUD9	132	Retrotransposable element
Q2PWB3	459	Gag-like protein
Q2PWB2	1000	Reverse transcriptase

Number of homologs and putative function of the gene families containing more than 10 members situated in the proximity of any of the 1085 TE1a homologs that were not associated to *AVR*_k1_ paralogs.

a Gene families defined by all homologs found with a cut-off E≤10^−5^. The names of the gene families correspond to the top hits.

### *AVR*_k1_ paralogs have coevolved with TE1a retrotransposons

The strong linkage between *AVR*_k1_ paralogs and the retroelement TE1a suggests a benefit to this association and, as a consequence, coevolution of the two genetic structures in the genome of *Bgh*. If two associated lineages coevolve, each lineage is expected to track the other over evolutionary time, which will be reflected in congruence between their phylogenies. Congruence between phylogenies of organisms is commonly ascribed to cospeciation in host-parasite systems [32], whereas incongruence is generally explained by events such as duplications, host-switch and parasite extinction. The equivalent processes for this genome analysis can be interpreted as codivergence instead of cospeciation, gene transfer within the genome instead of host-switch and gene loss instead of parasite extinction [33].

**Figure 3:**
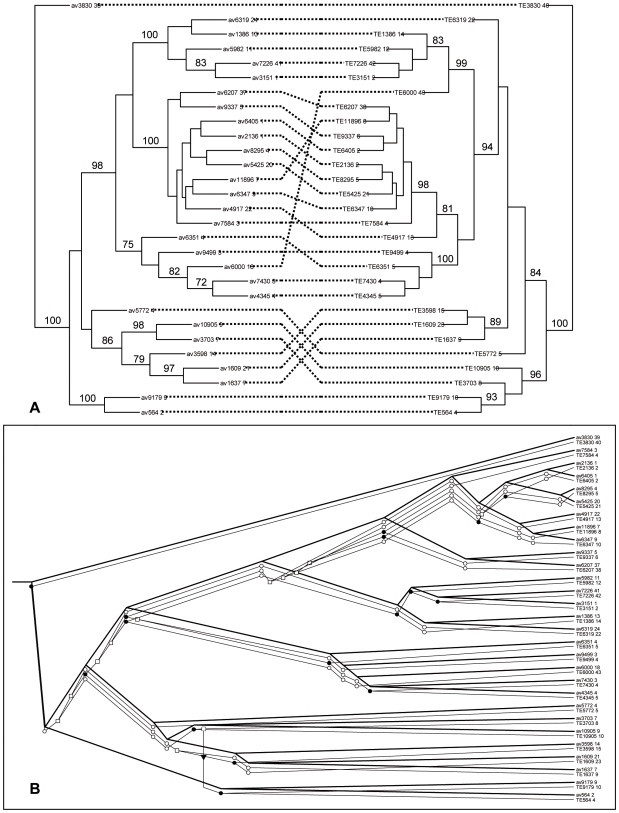
Comparison of the phylogenies of *AVR*_k1_ and TE1a sequences. A. Tanglegram for *AVR*_k1_ (left) and TE1a (right) sequences, based on predicted ORFs from *Bgh* genome. Lines connecting sequences indicate associations. Bootstrap support (1,000 replicates) is shown below the branch if higher than 70%. B. One of the four potentially optimal reconciled trees between *AVR*_k1_ and TE1a trees. The two trees are superimposed. Hypothetical evolutionary events are represented as black circles for codivergence events, white squares for duplication events, white circles for gene losses and arrows for gene transfers.

To explore the coevolutionary history of *AVR*_k1_ paralogs and TE1a sequences, we compared the phylogeny of these two groups by using the adjacent hits identified above. We used a mathematical model, Jungle [34], which contains all the combinations of associations between the two trees considering the events of codivergence, duplication, gene transfer and gene loss. We initially analyzed the 49 sequences that contained the entire conserved *AVR*_k1_ core sequence as previously defined [16], i.e. sequences that aligned to the central region of *AVR*_k1_, and were adjacent to a TE1a element. We applied cophylogenetic analysis to these 49 pairs of elements (Fig. S5) and then reduced the dataset to a more manageable subtree of 29 sequences that were selected because they form a large single clade in the larger tree (Fig. 3A). Two sub-clades of this group of *AVR*_k1_ sequences matched with similar clades in the TE1a phylogeny (subclades 1 and 4, Fig. S5). Since the computational complexity of the reconstruction problem is prohibitive when the number of gene transfers is large [34], we limited the Jungles reconciliation analysis to a maximum number of three gene transfers. Four potentially optimum solutions were identified: all four reconstructions postulated 32 codivergence events (equivalent to 16 instances of cospeciation) (Table 6, Fig 3B). The number of codivergence events was highly significant (P<0.01, the null hypothesis being the two phylogenies are randomly related) for scenarios with 0, 1 or 2 gene transfers, giving a good indication that *AVR*_k1_ and TE1a sequences have coevolved. However, the use of strong constraints (gene transfer ≤3) signifies a possible overestimation of the number of codivergence events and a probable underestimation of gene transfers.

**Table 6:** Codivergence between *AVR*_k1_ paralogs and TE1a sequences is highly significant.

Solution	Codivergences	Duplications	Gene transfers	Gene losses	Cost	P
1	32	24	0	55	79	<0.01
2	32	24	1	49	74	<0.01
3	32	24	2	44	70	<0.01
4	32	24	3	38	65	n/a

Costs of optimizations for co-divergence events in *AVR*_k1_/TE1a evolutionary reconstructions (illustrated in Fig 3A) are shown. The significance of each solution (P value) was determined by generating 99 random TE1a trees and calculating how many of the supported solutions included as many codivergence events as the observed *AVR*_k1_ tree. P values for solution 4 could not be calculated due to computational limitations.

We also used an event-based parsimony approach [35] to test the fit between the *AVR*_k1_ and TE1a phylogenies. This method finds the most likely explanation of observed data by minimizing the cost of implied events. We tested different reconstructions by preventing particular events from happening by applying a very high cost. We assigned a high cost to all four events in turn (codivergence, duplication, gene transfer and gene loss), and found a significant global fit between the two trees (P<0.001, the null hypothesis being the two phylogenies are randomly related) in all analyses, except when codivergence was prevented (P = 1), indicating that the similarity of *AVR*_k1_ and TE1 phylogenies is due to the number of codivergence events [36]. Using the same default values as in our first approach, we found that 10 to 12 codivergence events and 16 to 18 gene transfers maximize the likelihood of the model (P<0.001). These results indicate 1) a moderate fit between both phylogenies, and 2) that incongruences in the cophylogeny have most likely arisen by gene transfers from one genomic location to another. This means that the *AVR*_k1_ paralogs have coevolved with the TE1a sequences adjacent to them, although there have also been *AVR*_k1_ sequences that, in being transferred in the genome, have become close to TE1 retrotransposons with which they have not coevolved.

## Discussion

This work reveals that the *AVR*_k1_ family has extensively colonized the *Bgh* genome, representing the largest family of effector paralogs discovered so far in a fungal genome. A similar example of an extended number of related sequences within a given genome is the RXLR-containing effector family in oomycetes [30]. Functional redundancy of *AVR* genes within the genome may facilitate rapid evolution of the parasite to overcome host resistance by allowing elicitor genes to become inactivated without compromising parasite fitness [5], [37], [38]. The exceptionally high number of *AVR* genes described in *Bgh* [7] supports the idea of such an evolutionary history of this parasite.

*Blumeria* was the first genus that split from the rest of the Erysiphales 76 million years ago [39]. We found *AVR*_k1_ homologs in two *Erysiphe* species, so the gene family must predate the split. However, the *Erysiphe* sequences lie in the base of the phylogeny, not in the two large clades formed by *AVR*_k1_ or *AVR*_a10_ paralogs, so these subfamilies may have differentiated and proliferated extensively only in *Blumeria*. *AVR*_k1_ paralogs have evolved differentially in *B. graminis ff.spp*. from different grass hosts. The *AVR*_a10-_like sequences from ff. spp. *tritici*, *secalis* and *agropyri* group separately from those in ff. spp. *avenae*, *hordei* and the isolate from *Lolium perenne*. This corresponds with the phylogeny of other genes [40], in which isolates from ff. spp. *tritici*, *secalis* and *agropyri* form a distinct clade, with f.sp. *hordei* as a sister clade and ff. spp. *avenae* and isolates from *Lolium sp*. in more distantly related clades. Differential selection for a battery of effectors that are not recognized by the host could be the basis of host specialization of *B. graminis* [41]. Thus, it is possible that *AVR*_k1_ paralogs may be involved in the extreme host specialization encountered in this strictly biotrophic pathogen.

The selection pressure exerted on crops during the development of agriculture could have played an important role in promoting the proliferation and diversification of the *AVR*_k1_ family in *B. graminis*. After early cultivation of domesticated wheat, new powdery mildew resistance genes arose [42]. In the GFG system, mutation of the *AVR* genes would allow new, virulent isolates to escape recognition by these new resistance specificities. The greater abundance of *AVR*_k1_-like sequences in the *ff. spp*. from wheat, rye and barley, compared to those from oats, suggests that the proliferation of these genes could be related to the specialization of the parasite during the evolution of cereal crops in agriculture. Wheat, rye and barley originated in the near East during the 11th–9th millennia BP [43]. Oats originated much later as a crop in Northern Europe [4th–3rd millennia BP, 44], and have been subject to less intensive breeding than wheat and barley.

These data provide the first direct evidence that a parasite effector gene family and a particular retrotransposon lineage are consistently associated and have coevolved. The frequency with which members of the *AVR_k_*_1_ and TE1a retrotransposon lineages occur together in the genome is highly significant, and two independent analyses show that their phylogenies are congruent. The coevolution between these two entities indicates that they move and evolve together, so their occurrence close to each other is not merely due to a retrotransposon insertion site bias. An association with transposable elements has been postulated as a mechanism for the expansion and movement of effector genes within genomes [5], [6]. The coevolution of these two entities implies a mutual benefit to the association, which could ultimately contribute to parasite adaptation and success. The association would benefit 1) the powdery mildew fungi, by providing a mechanism for amplifying and diversifying effectors, which would increase the pathogen's mean fitness in the presence of diverse plant resistance genes and 2) the associated RTs, by providing a basis for their maintenance in the fungal genome through natural selection for genomes which contain numerous effector genes and thus contribute to increased fitness.

In addition to a role in gene mutation, RTs play an important role in genome evolution [45]–[47]. There is also considerable evidence that eukaryotic organisms have co-opted functions from RTs, including the epigenetic regulation of associated genes required for adaptation [48]. Such mechanisms could also apply to effectors, and be related to host adaptation [49]. We have found *AVR*_k1_ paralogs expressed as natural antisense transcripts (NATs) which can be a mechanism for epigenetic control of neighboring genes [31]. With an increasing number of genomes sequenced [50], it will be possible to establish whether coevolution between families of effectors and RTs occurs more widely, and how the association may contribute to parasite adaptation and host specialization.

In conclusion, we show that an effector gene family required for virulence in the powdery mildew fungus has coevolved with TE1a, a class of LINE-1 retrotransposon. To our knowledge, this is the first demonstration of the coevolution between parasite effectors and retrotransposons. An association between effectors and retrotransposons had already been postulated in many cases, but this is the first work that shows that this association is significant and has an evolutionary basis. Our discovery that effectors and retrotransposons have coevolved leads to a much deeper understanding of pathogenicity and specialization in parasites.

## Materials and Methods

### Fungal isolates and samples

Isolates of *Blumeria graminis* from different cultivated and wild grasses were obtained from the laboratory collection at the John Innes Centre. The *Bgh* isolate Race I [51] was used for making a cDNA library.

### RACE-PCR reactions

RNA was extracted with an RNAeasy kit (Qiagen) from leaves of barley cultivar Golden Promise, three days after inoculation with *Bgh* isolates A6, CC52, CC148, DH14 and from leaves of wheat cultivar Cerco, three days after inoculation with *B. graminis* f. sp. *tritici* (*Bgt*) isolate JIW11. Amplification of the 5′ and 3′ cDNA was performed with the SMART™ RACE kit (BD Biosciences). Twenty genomic sequences from a *Bgh* BAC library [16] were first obtained by hybridization to *AVR*_k1_. Primers were then designed to amplify expressed *AVR*_k1_ paralogs from four different *Bgh* isolates and a *Bgt* isolate. Following initial screening of primers to achieve the highest diversity in lengths for all the isolates, the primers used were: RACEK15′2 (5′AATGGCGGCGCGTAGGTAGACTCT3′) for the 5′end, nested with NESTEDK15′2 (5′CCCGTTGGTCAAAGGAAGAAGGGT3′) and RACE13′2 (5′TCGATGAGAGTCTACCTACGCGCC3′) for the 3′end, nested with NESTED15′2 (5′ATTGCGCAATACATGGCCACGGTG3′). Amplification products were cloned in the pGEM®-T Easy vector (Promega) and a random set of 24 clones per isolate were sequenced. The sequences have been deposited in the EMBL/GenBank [24], and accession numbers are GQ470737 to GQ470866.

### Sequencing of paralogs from different *ff. spp*

DNA was extracted as described previously [16] from conidia of *B. graminis* f. sp. *hordei* isolates DH14 and CC148; *tritici* isolates JIW11 and FEL09; *secalis* isolates RyeRMasBlue and RyeRmas6W; *avenae* isolates MO892 and MOH15; *agropyri* isolate CF3a. *B. graminis* and isolate LSSB1 from *L. perenne*. PCR was performed using AmpliTaq (Applied Biosystems) and degenerate PCR primers: AVRDEGF (5′GTCGARGCMRCCCTTCWWCC3′, where R = A+G, M = A+C, W = A+T) and AVRDEGR (5′GTGGCMCSWGTGCTTYTGAG3′, where Y = C+T, S = G+C). Sixteen to twenty six clones per isolate were sequenced. Only sequences with identities lower than 99% to any other sequence were considered as unisequences. The sequences have been deposited in the EMBL/GenBank [24], and accession numbers are GQ470682 to GQ470736.

### Isolation of cDNA clones

Full-length cDNA clones were isolated from a Lambda ZAP Express cDNA library [52], made from epidermal strips of barley leaves, cultivar Manchuria, 14–16 h after inoculation with *Bgh* isolate Race I [51]. The library was screened according to the ZAP Express manual (Stratagene) with a probe made from the conserved region of the *AVR*_k1_ gene family using the primers R1 and R3 [16] and 192 positive plaques were initially picked. From these, 22 clones were purified, *in vivo* excised and the inserts of the plasmids were sequenced. The sequences have been deposited in the EMBL/GenBank [24], and accession numbers are GQ470867 to GQ470888.

### Sequence analyses

Nucleotide sequence analysis and contig assembly were done with the STADEN package [53]. Protein sequences were aligned with MUSCLE [54] and edited with Genedoc (distributed by Nicholas KB, Nicholas HB and Deerfield DW, http://www.psc.edu/biomed/genedoc/gdfeedb.htm). Protein sequences were converted back to coding DNA sequences to conserve the codons position in the alignment using RevTrans [55]. Homologies were detected using the BLAST program [23] against the EMBL/GenBank [24], COGEME phytopathogen EST database [25], Broad Institute (http://www.broad.mit.edu/) and Uniprot [26] databases. Open reading frames were predicted from the draft genomes of *Bgh* (www.blugen.org), *Erysiphe* (*Golovinomyces*) *orontii* and *Erysiphe pisi* using the program getorf from the EMBOSS package [56].

Neighbor-Joining (NJ) and Maximum Likelihood trees were generated using the PHYLIP 3.6 package [57] and MEGA version 4 [58]. Distance matrices of the NJ trees were calculated under the Jones-Taylor-Thornton and the Jukes Cantor models of evolution for Figure 1 and Figure 2A respectively. Bootstrapping (100 or 1,000 replicates) was used to determine the strength of support for individual nodes. Likelihood mapping analyses [28] were done using the program TREE-PUZZLE 5.3 [59]. The dataset of sequences was classified in four groups under different hypotheses: a) depending on the host of origin (all possible combinations) and b) randomly. The posterior weights of the possible topologies of each quartet under each hypothesis were analyzed using the quartet puzzling algorithm.

The diversifying selection analyses were done using codeml from PAML 3.15 [60] with alignments of N-terminal and C-terminal regions. Two pairs of codon substitution models (M1a/M2a and M7/M8) were used to study ω variation among amino acid sites [61]. M1a and M7 assumes no site with ω >1 (no positive selection, null hypothesis) while M2a and M8 assumes the presence of positively selected sites. To test for positive selection, the likelihood ratio test (LRT) between the models in each pair was compared with a χ^2^ distribution. Whenever the LRT suggested the presence of positively selected sites, an empirical Bayes approach was used to calculate the conditional (posterior) probability distribution of ω for each site enabling the identification of positively selected residue in the alignment. Both Naive Empirical Bayes (NEB) and Bayes Empirical Bayes (BEB) methods were used [62].

In the cophylogenetic analysis, we compared *AVR*_k1_ and TE1a trees, using reconciliation analysis with Jungles [34] as implemented in the program TreeMap 2.0β. The analysis was performed with a maximum number of three host switches (or gene transfers). We used the default values for event costs: 0 for codivergence and 1 for duplication, loss and gene transfer (host switch) events. The significance of the codivergence events was determined by generating 99 random TE1a trees and determining how many of those supported solutions had as many codivergence events as the observed *AVR*_k1_ tree [63]. TreeFitter 1.0 [35] was used for parsimony-based tree fitting. The significance of the results was tested by performing 1,000 random permutations of the TE1a tree terminals.

### Sequences of *E. pisi* and *E. orontii*

*E. pisi* (Birmingham isolate, kindly provided by Dr. Timothy Carver from The Welcome Trust Sanger Institute, Hinxton, Cambridge, CB10 1SA, UK) and *E. orontii* (isolate MPIZ) genomic DNA was extracted from vacuum-harvested conidia and purified on a CsCl gradient. DNA sequencing by pyrosequencing (454 Technology) was performed by imaGenes, formerly RZPD German Resource Center for Genome Research in Berlin, Germany (http://www.imagenes-bio.de/) using GS-20 and FLX sequencer systems and automatically assembled on site. The available sequence corresponds to 400–450 Megabases each for *E. orontii* and *E. pisi* genomes.

## Supporting Information

Figure S1 Grouped likelihood mapping diagrams produced from the AVRa10 clade (Fig. 2A). A. The dataset was grouped in two clusters, a: agropyri - tritici - secalis and b: hordei - avenae - L. perenne. 91% of the quartets are (a,a) - (b,b), supporting the clusters defined. B. Sequences were randomly distributed in two clusters, a and b; any topology is favored. The analysis is consistent with the hypothesis that sequences from ff.spp. agropyri, tritici and secalis form a distinct clade in the phylogeny shown in Fig. 2A.
(0.99 MB TIF)

Figure S2 A. Diversifying selection at amino acid residues in AVRk1 homologs. Consensus representation of DS analysis on an alignment of RACE3′ or RACE5′ sequences. Sites were defined as diversified (in black) whenever the probability exceeds 90%. Otherwise, sites were defined as non-diversified (in grey). A residue with undefined adaptation (dotted) signifies discrepancy of results between the alignments of RACE3′ and RACE5′ sequences. Positions that were not analyzed are shown in white. The core sequence as defined in ref 16 is marked by dots above the sequence. Arrows show boundaries for 5′ and 3′ analysis. B. Breakpoints of divergence in expressed AVRk1 homologs. Representation of three full-length cDNA sequences obtained by hybridization to AVRk1, selected to illustrate how the sequence diverges after the conserved core region of AVRk1 (horizontal dotted line above the degree of homology to AVRk1). Sudden sequence divergence typically occurs in the break point region (shaded). Length of homology obtained by BLASTN against EMBL nucleotide database is shown by an horizontal line. Homologies identified by TBLASTX to expressed sequence tag (EST) of unknown function: * EST clone SL011D12–5, accession AU250405 from B. graminis-infected Lolium multiflorum.
(0.08 MB TIF)

Figure S3 A. Alignment of full-length cDNA sequences of AVRk1 paralogs from Fig. S2B showing sequence divergence breakpoint at arrow. B. Alignment of the other full-length cDNA sequences from Fig. S2B showing sequence divergence breakpoint at arrow.
(1.92 MB TIF)

Figure S4 Alignment of a natural antisense transcript (NAT) from two cDNA clones against the genomic sequence containing the AVRk1 sequence. Start of the AVRk1 coding sequence is highlighted in red. Conserved DNA sequence bases are indicated by an asterisk. The presence of poly dT at the 5′ end of the cDNA indicates polyadenylation of the transcript in the reverse orientation to that expected when compared to the AVRk1 sequence.
(1.06 MB TIF)

Figure S5 Tanglegram for AVRk1 (left) and TE1a (right) sequences, based on predicted ORFs from the Bgh genome. Lines connecting sequences indicate associations. Bootstrap support (100 replicates) is shown below the branch if higher than 70%. The groups of associated sequences selected for further analysis are numbered 1 to 4.
(0.93 MB TIF)
