# Supplementary figures and images for: Coevolution between a Family of Parasite Virulence Effectors and a Class of LINE-1 Retrotransposons
Source: PLoS One. 2009 Oct 15;4(10):e7463. doi: 10.1371/journal.pone.0007463 (PMC2759079; doi:10.1371/journal.pone.0007463)

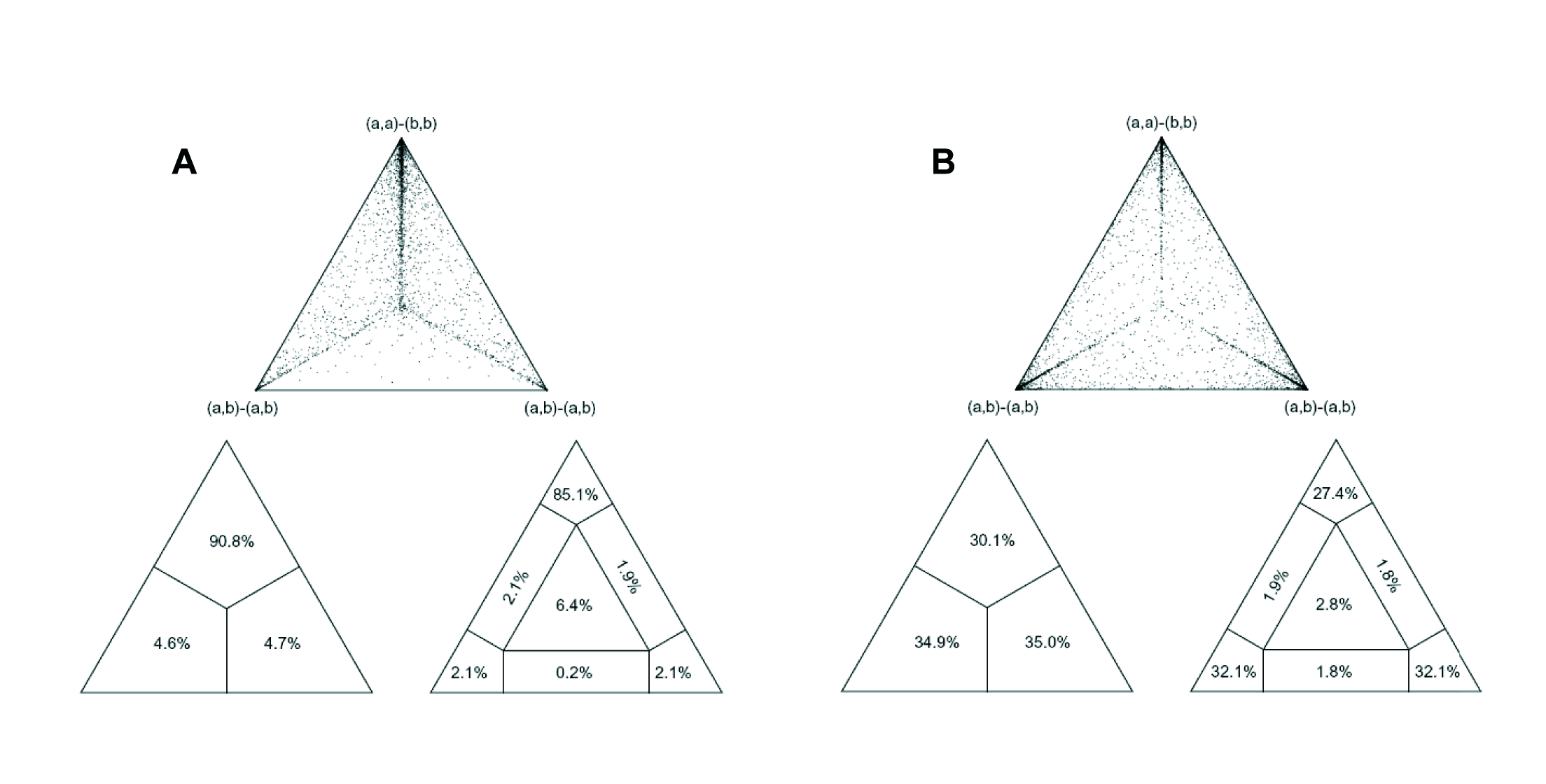

Supplement: Figure S1 — Grouped likelihood mapping diagrams produced from the AVRa10 clade (Fig. 2A). A. The dataset was grouped in two clusters, a: agropyri - tritici - secalis and b: hordei - avenae - L. perenne. 91% of the quartets are (a,a) - (b,b), supporting the clusters defined. B. Sequences were randomly distributed in two clusters, a and b; any topology is favored. The analysis is consistent with the hypothesis that sequences from ff.spp. agropyri, tritici and secalis form a distinct clade in the phylogeny shown in Fig. 2A. (0.99 MB TIF) [file pone.0007463.s001.tif]

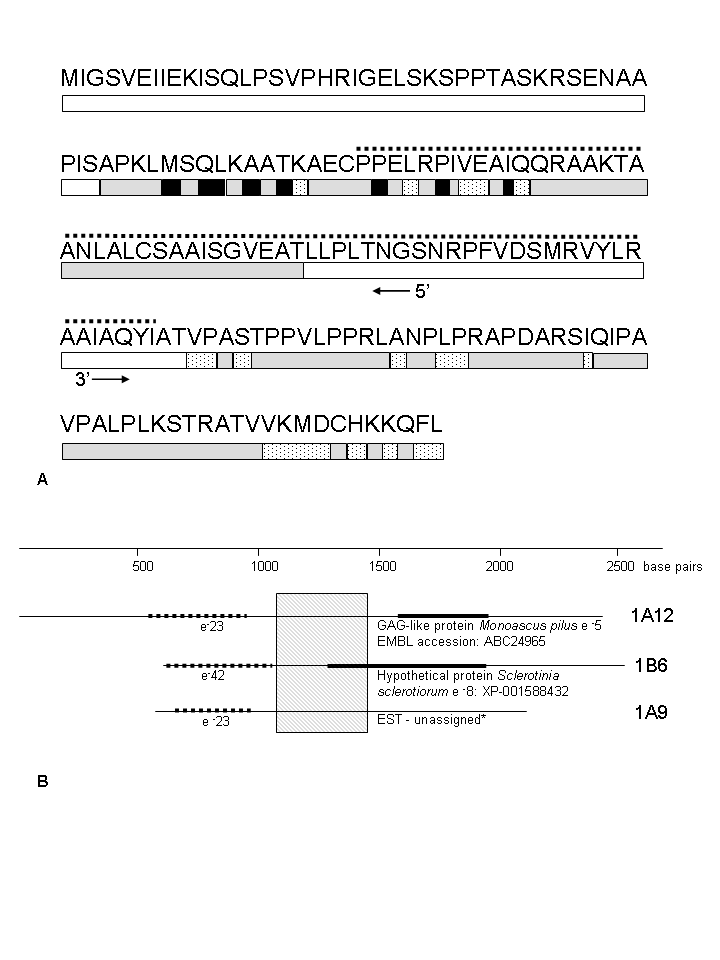

Supplement: Figure S2 — A. Diversifying selection at amino acid residues in AVRk1 homologs. Consensus representation of DS analysis on an alignment of RACE3′ or RACE5′ sequences. Sites were defined as diversified (in black) whenever the probability exceeds 90%. Otherwise, sites were defined as non-diversified (in grey). A residue with undefined adaptation (dotted) signifies discrepancy of results between the alignments of RACE3′ and RACE5′ sequences. Positions that were not analyzed are shown in white. The core sequence as defined in ref 16 is marked by dots above the sequence. Arrows show boundaries for 5′ and 3′ analysis. B. Breakpoints of divergence in expressed AVRk1 homologs. Representation of three full-length cDNA sequences obtained by hybridization to AVRk1, selected to illustrate how the sequence diverges after the conserved core region of AVRk1 (horizontal dotted line above the degree of homology to AVRk1). Sudden sequence divergence typically occurs in the break point region (shaded). Length of homology obtained by BLASTN against EMBL nucleotide database is shown by an horizontal line. Homologies identified by TBLASTX to expressed sequence tag (EST) of unknown function: * EST clone SL011D12–5, accession AU250405 from B. graminis-infected Lolium multiflorum. (0.08 MB TIF) [file pone.0007463.s002.tif]

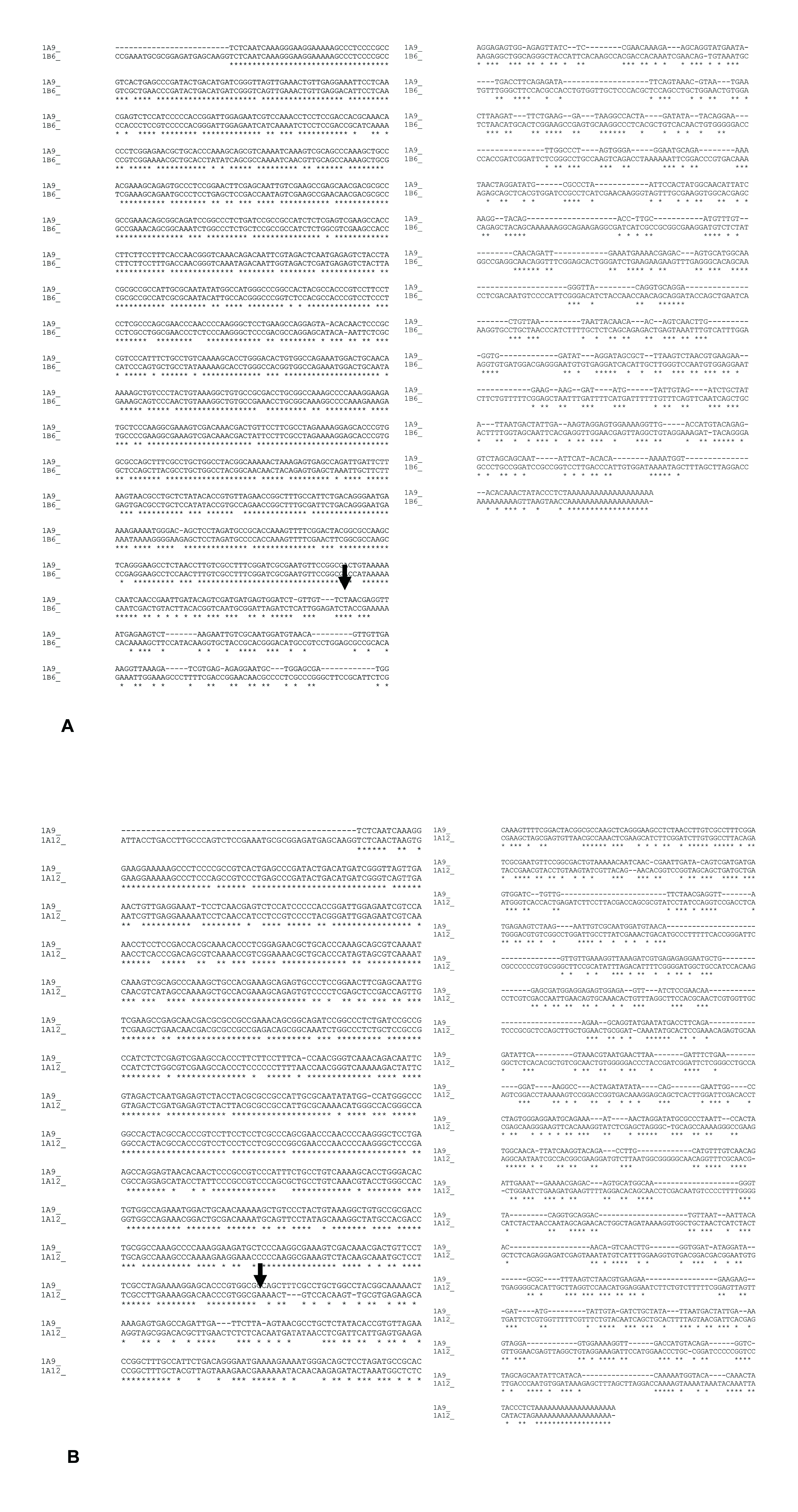

Supplement: Figure S3 — A. Alignment of full-length cDNA sequences of AVRk1 paralogs from Fig. S2B showing sequence divergence breakpoint at arrow. B. Alignment of the other full-length cDNA sequences from Fig. S2B showing sequence divergence breakpoint at arrow. (1.92 MB TIF) [file pone.0007463.s003.tif]

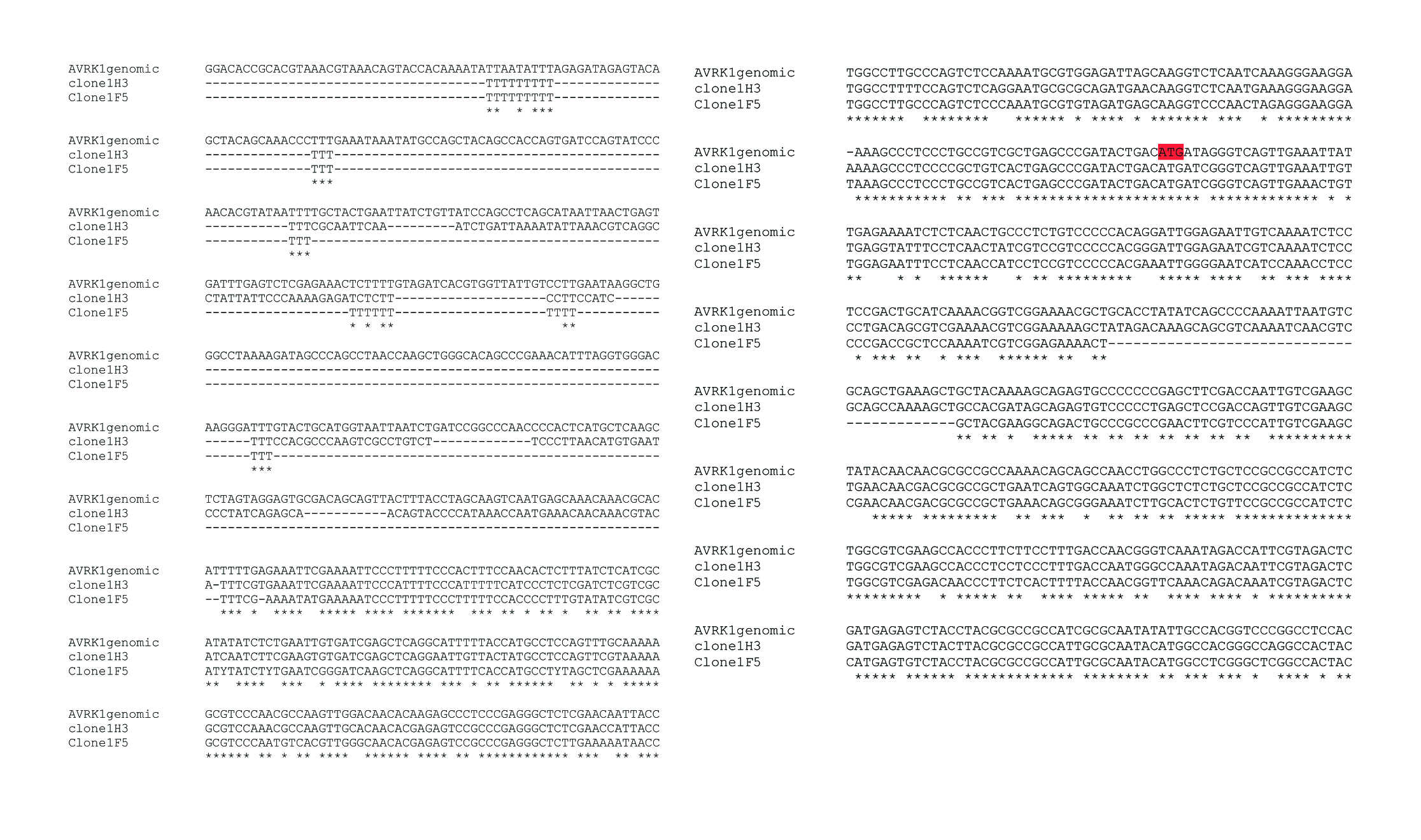

Supplement: Figure S4 — Alignment of a natural antisense transcript (NAT) from two cDNA clones against the genomic sequence containing the AVRk1 sequence. Start of the AVRk1 coding sequence is highlighted in red. Conserved DNA sequence bases are indicated by an asterisk. The presence of poly dT at the 5′ end of the cDNA indicates polyadenylation of the transcript in the reverse orientation to that expected when compared to the AVRk1 sequence. (1.06 MB TIF) [file pone.0007463.s004.tif]

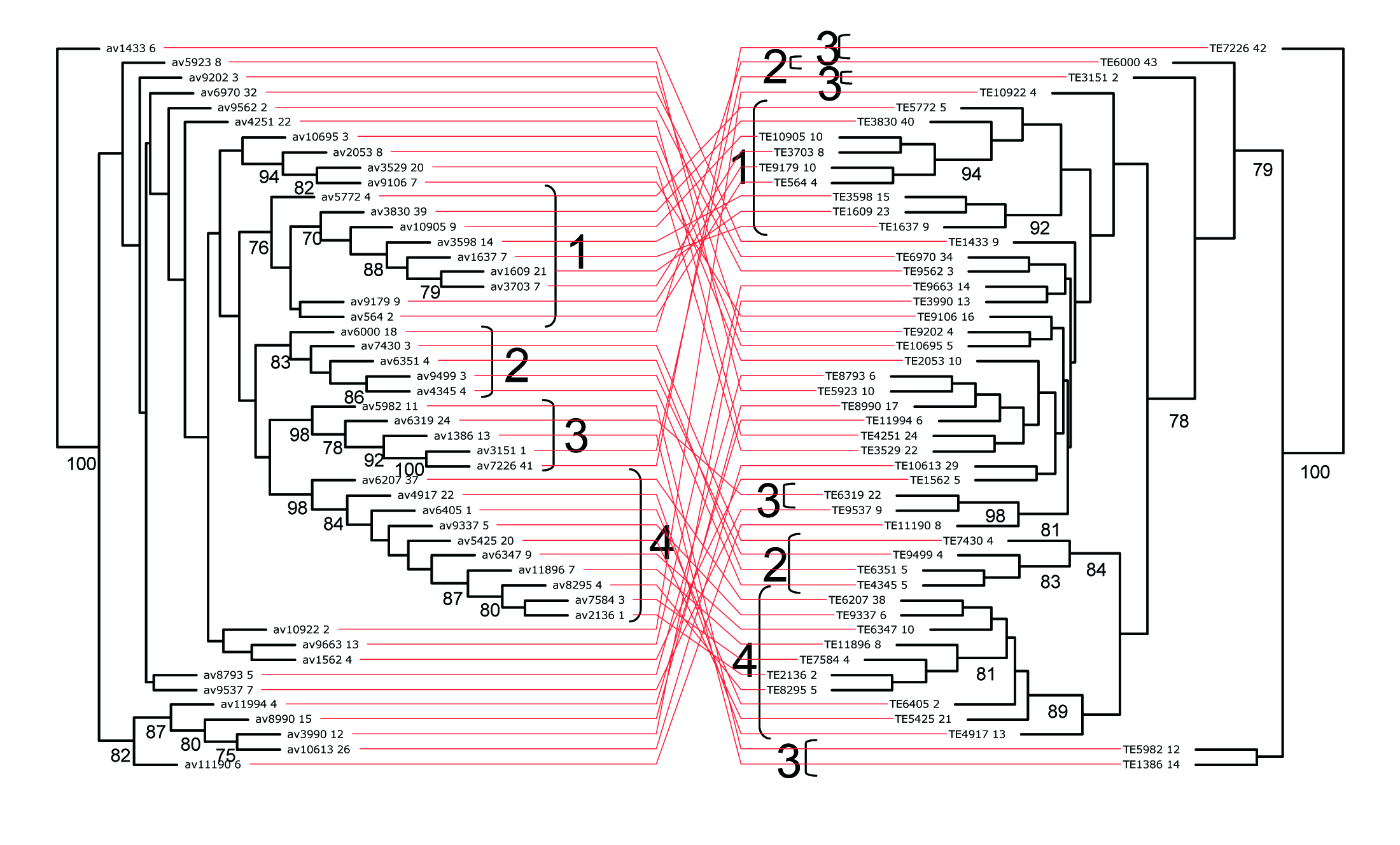

Supplement: Figure S5 — Tanglegram for AVRk1 (left) and TE1a (right) sequences, based on predicted ORFs from the Bgh genome. Lines connecting sequences indicate associations. Bootstrap support (100 replicates) is shown below the branch if higher than 70%. The groups of associated sequences selected for further analysis are numbered 1 to 4. (0.93 MB TIF) [file pone.0007463.s005.tif]
